# Nontraumatic osteonecrosis of the distal pole of the scaphoid

**DOI:** 10.4103/0019-5413.77142

**Published:** 2011

**Authors:** Bhavuk Garg, Himanshu Gupta, Prakash P Kotwal

**Affiliations:** Department of Orthopedics, All India Institute of Medical Sciences, New Delhi, India

**Keywords:** Scaphoid, AVN scaphoid, nontraumatic AVN scaphoid

## Abstract

Post traumatic osteonecrosis of distal pole of scaphoid is very rare. We present a case of 34 years old male, drill operator by occupation with nontraumatic osteonecrosis of distal pole of the scaphoid. The patient was managed conservatively and was kept under regular follow-up every three months. The patient was also asked to change his profession. Two years later, the patient had no pain and had mild restriction of wrist movements (less than 15 degrees in either direction). The radiographs revealed normal density of the scaphoid suggesting revascularization.

## INTRODUCTION

Osteonecrosis is one of the common complications of a scaphoid fracture. Its incidence has been reported to be ~10 – 15%,^1^,^2^ but approximately all patients with fracture involving the proximal fifth of the scaphoid progress to develop osteonecrosis.^2^,^3^ Osteonecrosis usually involves the proximal pole only. Although osteonecrosis of the whole scaphoid can be seen in the absence of trauma (Preiser’s disease), osteonecrosis of the distal pole of the scaphoid in the absence of trauma has not been reported to date. Only two published reports could be found in the literature reporting post traumatic osteonecrosis of only the distal pole of scaphoid.^4^,^5^ Another report described a case of osteonecrosis of both the poles of the scaphoid following a waist fracture in a 12-year-old boy.^6^

We present here a case of nontraumatic osteonecrosis of the distal pole of the scaphoid in a 34-year-old drill operator.

## CASE REPORT

**Figure 1:**
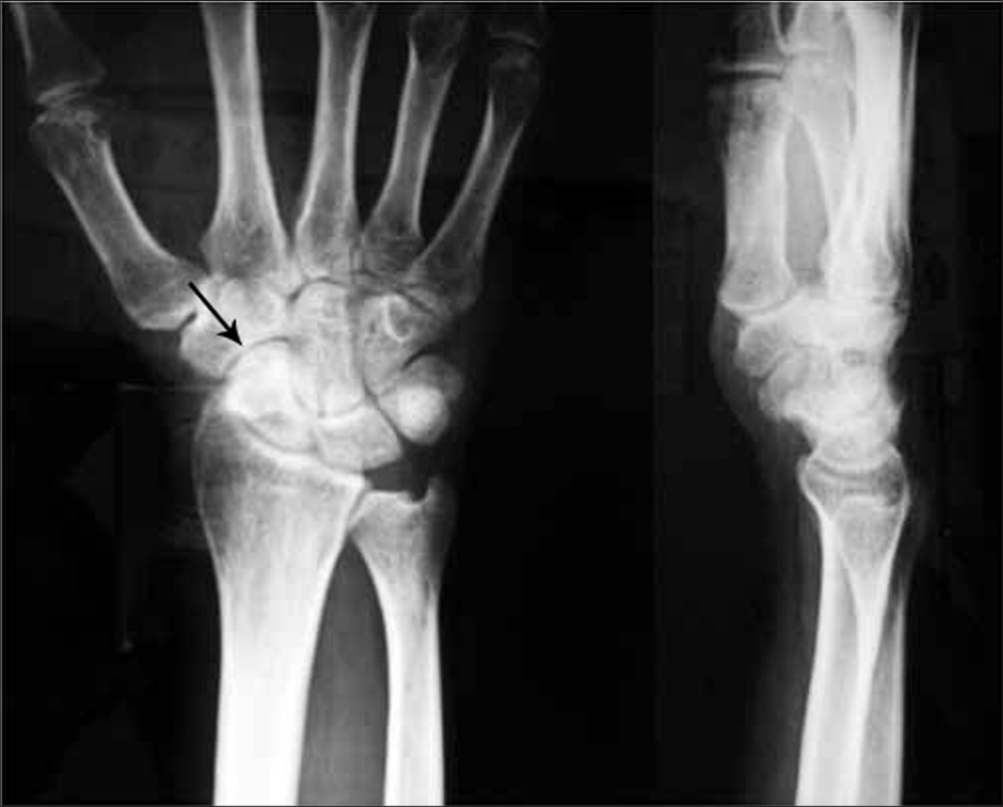
Anteroposterior and lateral radiograph of wrist showing increased density of distal pole of scaphoid (arrow) suggestive of avascular necrosis

**Figure 2:**
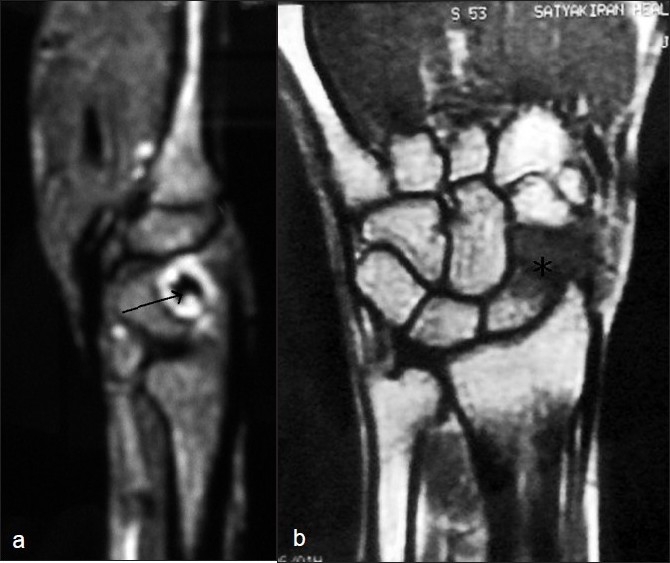
(a) MRI sagittal section through volar part of wrist showing osteonecrosis of distal pole of scaphoid (arrow): (b) MRI coronal section through mid part of the wrist showing osteonecrosis of distal pole of scaphoid (asterisk)

A 34-year-old male patient, a drill operator by occupation, presented to us with complaints of pain in the right wrist. The radiographs were not suggestive of any fracture or pathology and the patient was managed with analgesics for four weeks. The patient, however, complained of persistent pain. Four weeks later, the patient revisited us with complaints of persistent pain, along with restriction of activity. The patient was reinvestigated radiologically including radiographs [Figure 1] and magnetic resonance imaging (MRI) [Figure 2]. These showed osteonecrosis of the distal pole of the scaphoid, with low signal intensity of the distal pole in both the T1- and T2-weighted MRI images. No fracture was seen. The ligamentous structures were normal. The patient was managed conservatively and was kept under regular follow-up every three months. The patient was also asked to change his profession.

**Figure 3:**
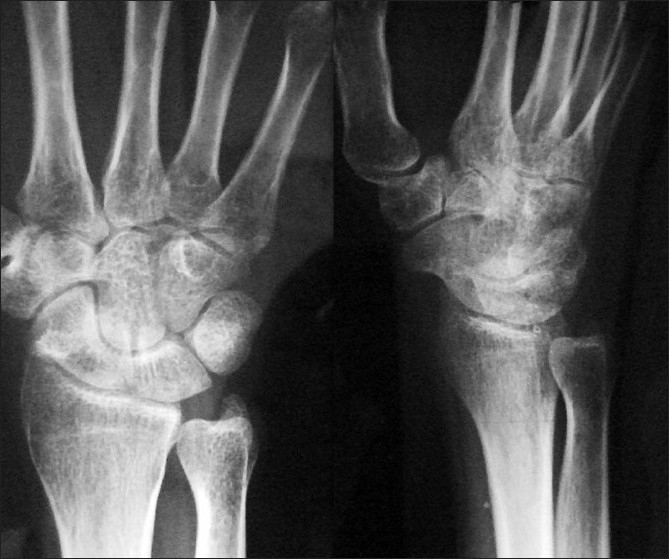
Two years later, anteroposterior and lateral radiograph showing reconstitution of normal architecture of scaphoid suggestive of revascularisation

Two years later, the patient had no pain and had mild restriction of wrist movements (less than 15 degrees in either direction). The radiographs revealed normal density of the scaphoid suggesting revascularization [Figure 3].

## DISCUSSION

Although osteonecrosis of the whole scaphoid can be seen in the absence of trauma (Preiser’s disease), osteonecrosis of the distal pole of scaphoid in the absence of trauma has not been reported to date. Osteonecrosis of the distal pole in our case may have occurred as a result of repeated microtrauma, leading to damage of all the dorsal vessels entering the bone distal to the waist. Cumulative micro trauma has been incriminated as a cause of osteonecrosis of other carpal bones such as lunate (Kienbock’s disease).^7^

Vibration exposure is another recognized cause of osteonecrosis of carpal bones.^7^,^8^ In fact, osteonecrosis of the scaphoid is recognized in many countries as an ‘occupational disorder caused by mechanical vibration’, entitling the patient for Worker’s Compensation.^9^ Vibration exposure is divided into two forms — whole body vibration and hand – arm vibration. The vibration in the latter group is transmitted through the handles or surface of a workpiece, via the palms and fingers. A number of occupational equipments have been incriminated, including drills, demolition hammers, angle grinder, chain saws, and handheld circular saws, to name a few. Musculoskeletal disorders around the wrist, which are thought to result from hand – arm vibration exposure include tendinitis, tenosynovitis, Dupuytren’s contracture, carpal tunnel syndrome, osteonecrosis of carpal bones (lunate, scaphoid, os triquetrum, capitate), cysts and vacuoles, and osteoarthrosis of the radiocarpal, intercarpal and distal radioulnar joint.^7^–^10^ Osteonecrosis is a well-known complication of the Scaphoid fractures.^1^,^2^ However, it usually inflicts the proximal pole only, and the lucid description of the vascularity of the scaphoid by Gelberman **et al**.^11^ explains the reason for this predilection for the proximal pole. The scaphoid derives its blood supply from the radial artery via two groups of vessels. The volar group palmar scaphoid vessels arise from the radial artery 75% of people, and from the superficial palmar branch of the radial artery in the remainder of the people. It enters and supplies the tubercle to account for the distal 20 – 30% of the intraosseous vascularity. The proximal 70 – 80% of the vascularity comes from vessels penetrating the bone along an obliquely oriented ridge on the dorsal surface of the bone. These vessels arise either from the radial artery (70%) or from the intercarpal artery (23%). In 7%, the scaphoid receives its dorsal blood supply directly from the branches of both these arteries. After entering the bone at the ridge, these vessels run proximally to supply the proximal pole. The scaphoid receives no vascular contribution from the scapholunate ligament, and there are no apparent anastomoses between the palmar and the dorsal vasculatures.^2^,^11^–^13^

A fracture running proximal to the insertion of the dorsal ridge vessels, or one which damages these vessels predisposes the vascularity of the proximal fragment. The site of entry of the dorsal vessels is variable — they enter distal to the waist in 14%, at the waist in 79%, and proximal in 7%.^13^ Thus, in the rare event of all the dorsal vessels entering the bone proximal to the waist, a fracture through the waist might possibly lead to osteonecrosis of the distal fragment if (1) the volar vessels get damaged at the time of injury, or (2) there is an anomalous deficient vascularization of the distal pole. In either case, the probability seems to be rare, as is evident from the paucity of reports in the literature.^4^,^5^

Avascular necrosis of the scaphoid has been reported in patients with collagen vascular disease.^14^ There were no clinical symptoms or signs pointing toward the same. There was no history of any prolonged drug intake including steroids or chemotherapy.

Osteonecrosis of the distal pole in our case probably occurred as a result of repeated microtrauma leading to a damage of all the dorsal vessels entering the bone proximal to the waist. A change of profession and conservative management has given good results in our case.
